# Dissolving sodium hydrosulfide in drinking water is not a good source of hydrogen sulfide for animal studies

**DOI:** 10.1038/s41598-023-49437-y

**Published:** 2023-12-09

**Authors:** Asghar Ghasemi, Sajad Jeddi, Nasibeh Yousefzadeh, Khosrow Kashfi, Reza Norouzirad

**Affiliations:** 1 Endocrine Physiology Research Center, Research Institute for Endocrine Sciences, Shahid Beheshti University of Medical Sciences, Tehran, Iran; 2 Department of Molecular, Cellular, and Biomedical Sciences, Sophie Davis School of Biomedical Education, City University of New York School of Medicine, New York, NY USA; 3 Department of Biochemistry, School of Medicine, Dezful University of Medical Sciences, Dezful, Iran

**Keywords:** Biochemistry, Physiology

## Abstract

Hydrogen sulfide (H_2_S) has many physiological and pathological roles in the human body. Sodium hydrosulfide (NaHS) is widely used as a pharmacological tool for assessing H_2_S effects in biological experiments. Although H_2_S loss from NaHS solution is a matter of minutes, some animal studies use NaHS in solution as an H_2_S-donating compound in drinking water. This study addresses whether 30 μM NaHS in drinking water prepared in rat/mouse water bottles remains stable for at least 12–24 h, as presumed by some authors. NaHS solutions (30 μM) were prepared in drinking water and immediately transferred to rat/mice water bottles. Samples were obtained from the tip of water bottles and from inside of the bottles at 0, 1, 2, 3, 4, 5, 6, 12, and 24 h for sulfide measurement using the methylene blue method. Furthermore, NaHS (30 μM) was administered to male and female rats for two weeks, and serum sulfide concentrations were measured every other day in the first week and at the end of the second week. NaHS solution was unstable in the samples obtained from the tip of water bottles; it declined by 72% and 75% after 12 and 24 h, respectively. In the samples obtained from the inside of the water bottles, the decline in the NaHS was not significant until 2 h; however, it decreased by 47% and 72% after 12 and 24 h, respectively. NaHS administration did not affect serum sulfide levels in male and female rats. In conclusion, NaHS solution prepared in drinking water can not be used for H_2_S donation as the solution is unstable. This route of administration exposes animals to variable and lower-than-expected amounts of NaHS.

## Introduction

The history of hydrogen sulfide (H_2_S) as a toxin dates back to 1700^1^; however, its possible role as an endogenously-produced biological signaling molecule was reported in 1996 by Abe and Kimura^2^. In the past three decades, many functions of the H_2_S in various human systems were elucidated^1,3^, leading to the recognition that H_2_S-donating molecules might have clinical utility in the treatment or management of some diseases^3,4^; see Cirino et al.^3^ for a recent review.

In many cell culture and animal studies, sodium hydrosulfide (NaHS) is widely used as a pharmacological tool for assessing H_2_S effects^5–8^. However, NaHS is not an ideal H_2_S donor because of its fast conversion to H_2_S/HS^-^, contamination with polysulfides, oxidation, and volatilization when in solution^4,9^. In many biological experiments, NaHS is dissolved in water, resulting in passive volatilization and H_2_S loss^10–12^, spontaneous H_2_S oxidation^11–13^, and photodecomposition^14^. Sulfide loss from stock solutions via H_2_S volatilization is fast^11^, and in open chambers, H_2_S is lost with a t_1/2_ of about 5 min, where its concentration falls by approximately 13% per minute^10^. Although H_2_S loss from a NaHS solution is a matter of minutes, some animal studies have used NaHS solutions in drinking water as an H_2_S source for 1–21 weeks, changing the NaHS-containing solution every 12–24 h^15–26^. This practice is incompatible with scientific research principles as the drug dose should be defined to translate it to other species, particularly humans^27^.

Preclinical studies in biomedicine aim to improve patient care or health. However, the results of most animal studies are not translated to humans^28–30^. One cause for this failure in translation is paying less attention to the methodological quality of animal studies^30^. Therefore, this study addresses whether 30 μM NaHS solutions in drinking water prepared in rat/mouse water bottles remain stable for 12–24 h, as claimed or presupposed in some studies.

## Materials and methods

### Ethical approval

All experiments of the current study were affirmed by the published guidelines for the care and use of laboratory animals in Iran^31^. All experiments of the current study were also reported following ARRIVE guidelines^32^. The ethics committee of the Research Institute for Endocrine Sciences, affiliated with the Shahid Beheshti University of Medical Sciences, confirmed and approved all experimental procedures of the current study.

### Materials

Zinc acetate dihydrate (CAS: 5970-45-6) and ferric chloride anhydrous (CAS: 7705-08-0) were purchased from Biochem, Chemopahrama (Cosne Sur Loire, France). Sodium hydrosulfide hydrate (CAS: 207683-19-0) and N,N-dimethyl-p-phenylenediamine (DMPD) (CAS: 535-47-0) were purchased from Sigma-Aldrich (Saint Louis, MO, USA). Isoflurane was purchased from Piramal (Bethlehem, PA, USA). Hydrochloric acid (HCl) was obtained from Merck (Darmstadt, Germany).

### Preparation of NaHS solutions

NaHS solutions (30 μM) was prepared in drinking water and immediately transferred to rat/mice water bottle. This concentration was chosen based on many publications that use NaHS as an H_2_S source in their studies; please see the discussion section. NaHS is a hydrated molecule and may have variable numbers of waters of hydration (i.e., NaHS•xH_2_O); according to the manufacturer, the percent of NaHS used in our study was 70.7% (i.e., NaHS•1.3 H_2_O) and this value was considered in our calculations, where we used a molecular weight of 56.06 g/mol, which is for anhydrous NaHS. Waters of hydration (also called waters of crystallization) are water molecules integral to the crystal structure^33^. Hydrates have different physical and thermodynamic properties compared to anhydrates^34^.

The solvent's pH and temperature were measured before adding the NaHS to the drinking water. NaHS solution was immediately transferred to the water bottles of rats/mice in the animal cages. Samples were obtained from the tip of water bottles and from inside of the bottles at 0, 1, 2, 3, 4, 5, 6, 12, and 24 h for sulfide measurement. Sulfide measurement was done immediately after each sampling. We obtained samples from the tip of the bottles as some studies have stated that the small aperture of water bottles minimizes H_2_S evaporation^15,19^. This issue seems to be true for the solution inside the bottle. However, this is not the case for solution in the tip of water bottles with a higher evaporation rate and auto-oxidation; indeed, animal drinks this water first.

### Experiments in rats

In this study male and female Wistar rats were used. Rats were housed in polypropylene cages (2–3 rats/cage) under standard conditions (temperature 21–26 °C and humidity 32–40%) of 12-h light (7 am to 7 pm) and 12-h dark (7 pm to 7 am). They had free access to tap water and the regular chow diet (Khorak Dam Pars, Co., Tehran, Iran). Age-matched (6-month old) female (n = 10, body weight: 190–230 g) and male (n = 10, body weight: 320–370 g) Wistar rats were randomly assigned to control and NaHS (30 μM)-treated groups (n = 5/group). We used the KISS (Keep It Simple, Stupid) approach to determine sample size, which combines past experience with power analysis^35^. We first performed a pilot study with 3 rats and determined the mean total serum sulfide levels and standard deviation (8.1 ± 0.81 μM). Then, considering 80% power and assuming a two-sided 5% significance level, we determined our provisional sample size (n = 5 based on previous literature) that corresponded to a standardized effect size of 2.02 from predefined values presented by Festing for sample size calculation in laboratory animals^35^. After multiplying this value by SD (2.02 × 0.81), the predicted detectable effect size (1.6 μM) was 20%, which is acceptable. It means that n = 5/each group is sufficient to detect a mean change of 20% between groups. Randomization of rats to control and NaSH-treated groups was done using the random function of the Excel software^36^ (Supplementary Fig. 1). Blinding was done at the outcome level, and the researcher who performed biochemical measurements was blinded to the groups.

NaHS groups of both sexes were treated with 30 μM of NaHS solution prepared in drinking water for 2 weeks; fresh solutions were provided every 24 h, at which time body weights were measured. Blood samples were obtained from the tail tips of all rats under isoflurane anesthesia every other day in the first week and at the end of the second week. Blood samples were centrifuged at 3000 *g* for 10 min, and sera were separated and maintained at – 80 °C for subsequent measurement of serum urea, creatinine (Cr), and total sulfide. Serum urea was measured by the enzymatic urease method and serum Cr by the photometric Jaffe method using commercially available kits (Man Company, Tehran, Iran) and an auto-analyzer (Selectra E, serial number 0–2124, Netherlands). Intra- and inter-assay coefficient of variation for both urea and Cr were < 2.5%.

### Total sulfide measurement

The methylene blue (MB) method was used to measure total sulfide in NaHS-containing drinking water and serum; MB is the most commonly used method for sulfide measurement in stock solutions and biological samples^11,37^. The MB method is useful for estimating total sulfide pools^38^ and measures inorganic sulfide present as H_2_S, HS^-^, and S^2^ in the aqueous phase^39^. In this method, sulfur is precipitated as zinc sulfide (ZnS) in the presence of zinc acetate^11,38^. Precipitation by zinc acetate is the most widely used technique for separating sulfide from other chromophores^11^. ZnS is redissolved under highly acidic conditions using HCl^11^. The sulfide reacts with DMPD in ferric chloride (Fe^3+^ acts as an oxidizing agent) catalyzed reaction with 1:2 stoichiometric ratio to give the MB dye, which is detected spectrophotometrically at 670 nm^40,41^. The detection limit of the MB method is around 1 µM^11^.

In the current study, 100 μL of each sample (solution or serum) was added to a test tube; then, 200 μL of zinc acetate (1% w/v in distilled water), 100 μL of DMPD (20 mM in HCl 7.2 M), and 133 μL of FeCl_3_ (30 mM in HCl 1.2 M) were added. The mixture was incubated in a dark environment at 37ºC for 30 min. The solution was centrifuged at 10,000 *g* for 10 min, and the absorbance of the supernatant was read at 670 nm using a microplate reader (BioTek, MQX2000R2, Winooski, VT, USA). A NaHS (0–100 μM) calibration curve in ddH2O was used to determine sulfide concentrations (Supplementary Fig. 2). All solutions used for measurement were prepared freshly. Intra- and inter-assay coefficients of variation of sulfide measurement were 2.8% and 3.4%, respectively. Using the spiked samples method, we also determined the total sulfide recovered for NaSH-containing drinking water and serum samples^42^. Recovery values for NaSH-containing drinking water and serum samples were 91 ± 1.1% (n = 6) and 93 ± 2.4% (n = 6), respectively.

### Statistical analyses

GraphPad Prism version 8.0.2 for Windows (GraphPad Software, San Diego, California USA, www.graphpad.com) was used for statistical analyses. The temperature and pH of drinking water before and after adding NaHS were compared using a paired t-test. Loss of H_2_S from NaHS-containing solutions was calculated as the percent decrease from baseline absorbance, and to assess whether this loss is statistically significant, one-way repeated measure ANOVA followed by the Dunnett multiple comparison test was used. Two-way mixed (between-within) ANOVA with the Bonferroni posthoc test was used for comparing body weight, serum urea, serum Cr, and serum total sulfide between control and NaHS-treated rats over time in each sex. Two-sided P-values < 0.05 were considered statistically significant.

## Results

### Stability of NaHS solution

**Figure 1:**
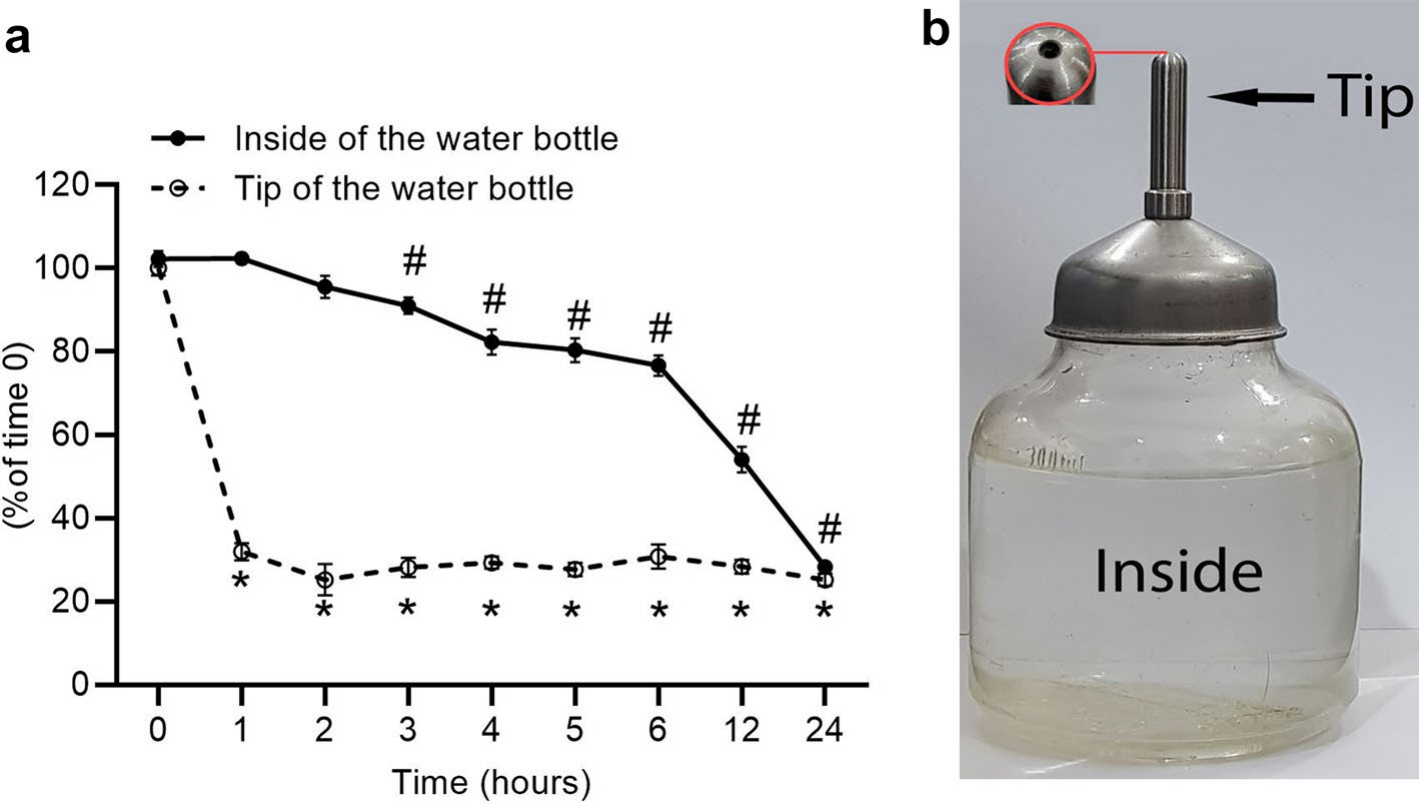
Stability of NaHS solution (30 μM) in drinking water maintained in rat/mice water bottle. After the solution was prepared, samples were obtained from the tips of the water bottles and the inside. Data are mean ± SE (n = 6/group). * and #, P < 0.05 compared to time 0. Picture of water bottle indicating the tip (with its aperture) and body. The volume of the tip is about 740 μL.

The pH of the drinking water was 7.60 ± 0.01 before the addition of the NaHS and 7.71 ± 0.03 (n = 13, p = 0.0029) after adding NaHS. The drinking water temperature was 26.5 ± 0.2, which decreased to 26.2 ± 0.2 (n = 13, p = 0.0128) after adding NaHS. 30 μM solutions of NaHS were prepared in drinking water and maintained in the water bottles. The NaHS solution was unstable, and its concentration declined with time. When samples were obtained from the tip of the water bottles, a large decrease (68.0%) was observed during the first hour, and the amount of NaHS in the solution declined by 72% and 75% after 12 and 24 h, respectively. In the samples obtained from the inside of the water bottles, the decline in the NaHS was not significant until 2 h; however, it decreased by 47% and 72% after 12 and 24 h, respectively. These data indicate that regardless of the sampling location, the percent of NaHS in a 30 μM solution prepared in drinking water is decreased to about one-fourth of the initial value after 24 h (Fig. 1).

### Changes in NaHS concentration of drinking water

**Figure 2:**
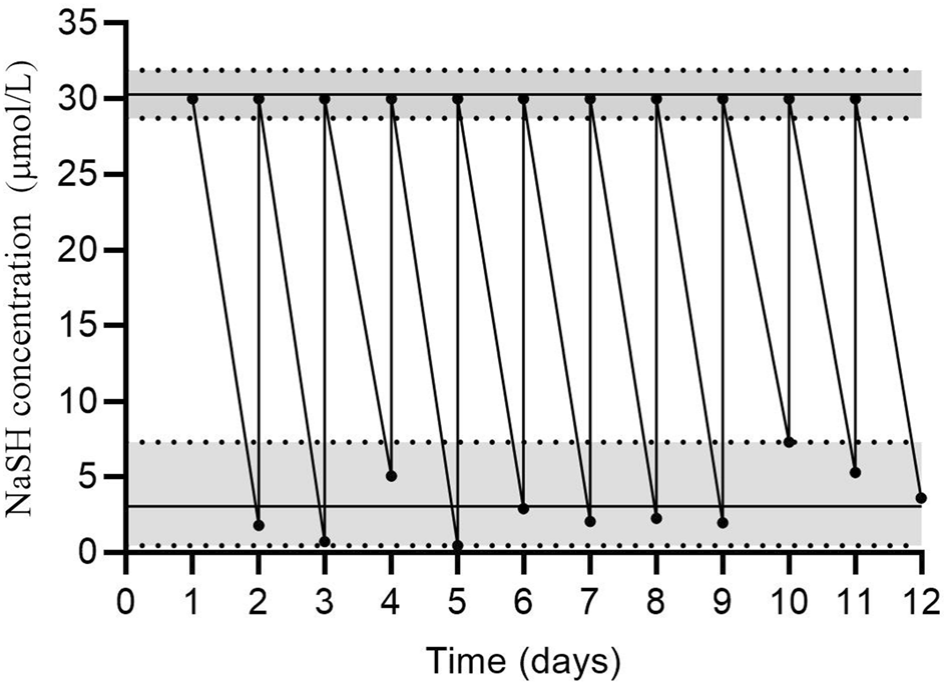
Changes in NaHS concentrations of drinking water to which rats were exposed during the study.

The concentration of NaHS in freshly prepared 30 μM solutions was 30.3 ± 0.4 μM (range: 28.7–31.9 μM, n = 12). However, it declined to low values (mean: 3.0 ± 0.6 μM) after 24 h. As shown in Fig. 2, rats were not exposed to a stable concentration of NaHS during the study.

### Effect of NaHS administration on body weight, serum total sulfide, and kidney function in rats

**Figure 3:**
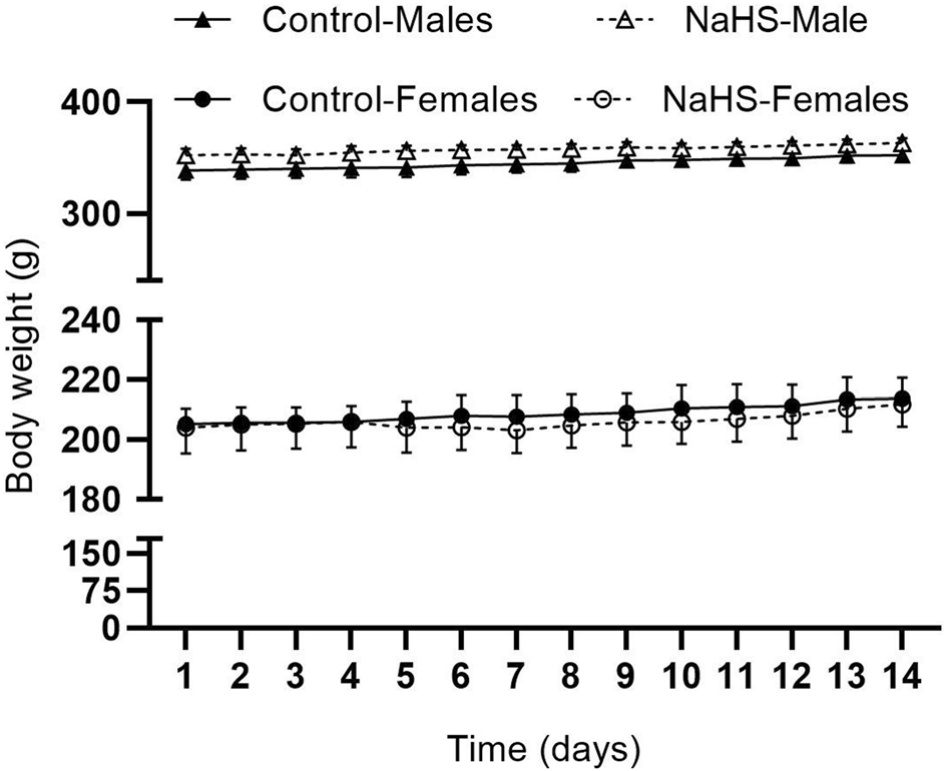
Changes in body weight following administration of NaHS (30 μM) in female and male rats. Data are mean ± SEM and were compared using two-way mixed (between-within) ANOVA with the Bonferroni posthoc test. n = 5/group in each sex.

Body weight of female rats increased significantly over time (from 205.2 ± 5.2 to 213.8 ± 7.0 in control and from 204.0 ± 8.6 to 211.8 ± 7.5 g in NaHS-treated rats); however, NaHS administration did not affect body weight (Fig. 3). Body weight of male rats increased significantly over time (from 338.6 ± 8.3 to 352.4 ± 6.0 in control and from 352.4 ± 5.9 to 363.2 ± 4.3 g in NaHS-treated rats); however, NaHS administration did not affect body weight (Fig. 3).

Serum urea and Cr concentrations were comparable in control and NaSH-treated rats throughout the study. In addition, NaSH treatment did not affect serum urea and Cr concentrations (Table 1).

**Table 1:** Changes in serum urea and creatinine (Cr) (mg/dL) of male and female rats in control and NaSH-treated (30 μM) (NaSH) groups at different time points.

	Time points (days)									
	0		2		4		6		14	
	Control	NasH	Control	NasH	Control	NasH	Control	NasH	Control	NaSH
Male										
Urea	52.0 ± 1.7	51.2 ± 0.9	52.0 ± 2.4	50.6 ± 1	51.2 ± 0.6	49.6 ± 0.9	49.2 ± 1.2	51.0 ± 2.2	51.6 ± 0.8	51.2 ± 1.2
Cr	0.88 ± 0.07	0.90 ± 0.03	0.86 ± 0.04	0.94 ± 0.05	0.96 ± 0.07	0.84 ± 0.05	0.90 ± 0.07	0.80 ± 0.01	0.88 ± 0.06	0.92 ± 0.06
Female										
Urea	53.0 ± 0.3	52.4 ± 0.7	50.0 ± 1.1	50.6 ± 1.6	49.6 ± 1.3	50.8 ± 0.9	51.5 ± 0.6	50.6 ± 2.3	49.3 ± 1.9	50.2 ± 0.7
Cr	0.84 ± 0.02	0.84 ± 0.02	0.86 ± 0.02	0.84 ± 0.05	0.96 ± 0.04	0.90 ± 0.08	0.80 ± 0.04	0.82 ± 0.02	0.88 ± 0.09	0.86 ± 0.02

Data are mean ± SEM and were compared using two-way mixed (between-within) ANOVA with the Bonferroni posthoc test. n = 5/group in each sex.

**Figure 4:**
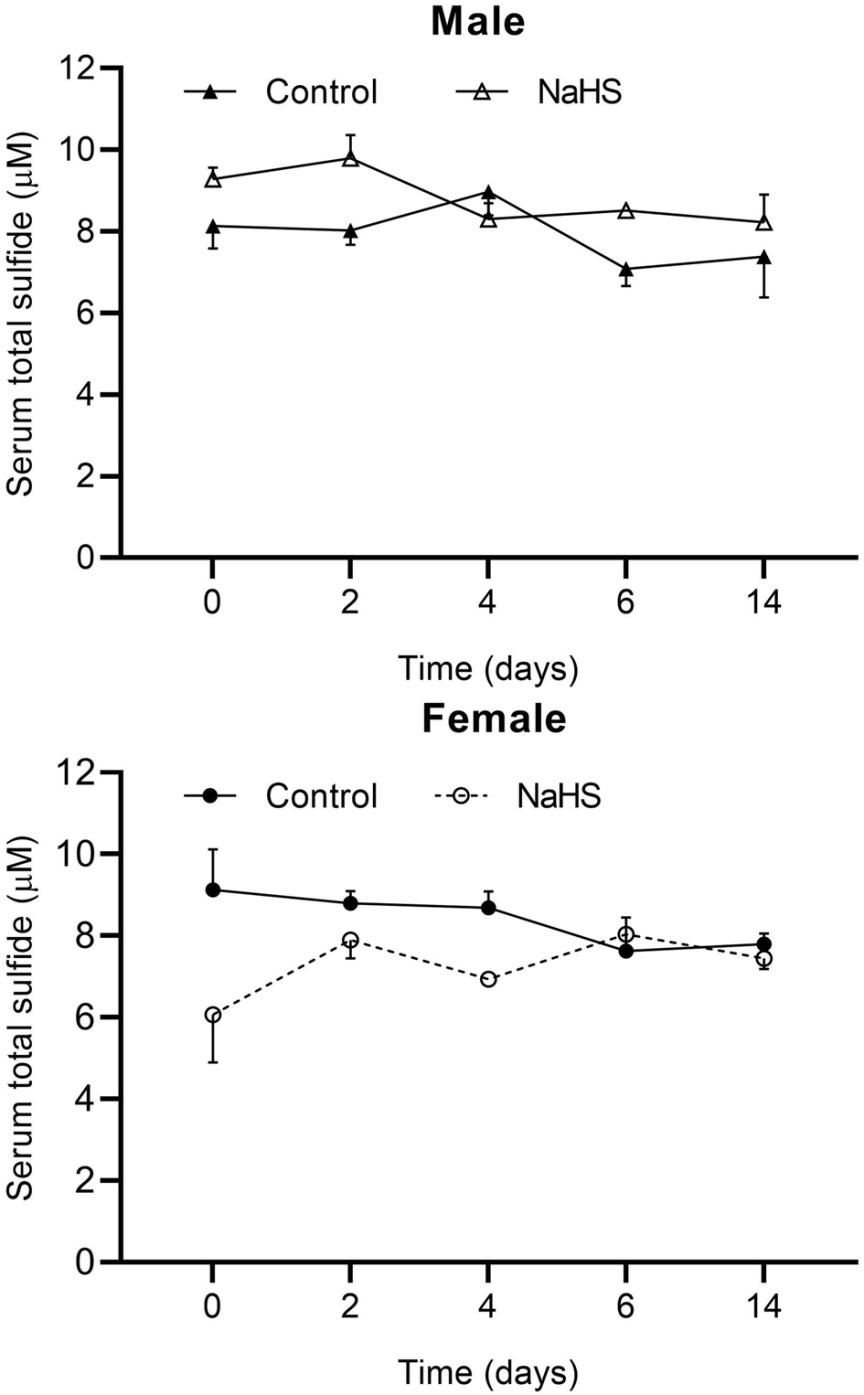
Changes in serum total sulfide concentrations following administration of NaHS (30 μM) in female and male rats. Data are mean ± SEM and were compared using two-way mixed (between-within) ANOVA with the Bonferroni posthoc test. n = 5/group in each sex.

Baseline serum total sulfide concentrations were comparable in control and NaHS-treated male (8.1 ± 0.5 vs. 9.3 ± 0.2 μM) and female (9.1 ± 1.0 vs. 6.1 ± 1.1 μM) rats. NaHS administration over 14 days did not affect serum total sulfide levels in male and female rats (Fig. 4).

## Discussion

The main result of this study is that NaHS-containing drinking water is not stable, and only about one-fourth of the baseline total sulfide was recovered after 24 h following sampling from the tip and inside of rat/mice water bottles. In addition, due to H_2_S loss from the NaHS solution, rats were not exposed to a stable concentration of NaHS, and NaHS administration in drinking water did not affect body weight, serum urea and Cr, and serum total sulfide levels.

In this study, the rate of H_2_S loss was about 3% per hour of 30 μM NaHS solutions prepared in drinking water. Time-dependent decrease in sulfide concentration has been reported to be 7% per hour in buffer solutions (sodium sulfide, 100 μM in 10 mM PBS, pH = 7.4) over 8 h^11^. We previously, in defense of intraperitoneal injection of NaHS, reported that the sulfide loss rate from NaHS-containing drinking water in a 54 μM solution was about 2.3% per hour (4%/hour for the first 12 h and about 1.4%/hour for the second 12 h after its preparation)^8^. The steady loss of H_2_S from NaHS solution has been emphasized in earlier works^43^ and is mainly done via volatilization and oxidation. Even without bubbling, sulfide loss from stock solutions via H_2_S volatilization is fast^11^. It has been shown that during dilution of stock solution, which took about 30–60 s, about 5–10% of H_2_S is lost by evaporation^6^. To avoid H_2_S evaporation from the solutions, researchers have adopted some measures, including gentle mixing of solutions^12^, covering the stock solutions with parafilm^6^, and minimizing exposure of the solutions to air, as the rate of H_2_S evaporation depends on the air–liquid interface^13^. Spontaneous oxidation of H_2_S is primarily due to transition metal ions, particularly ferric iron, which exists as an impurity in water^13^. Oxidation of H_2_S causes the formation of polysulfides (sulfur atoms connected with covalent bonds)^11^. To avoid its oxidation, H_2_S-containing solutions are prepared in deoxygenated solvents^44,45^ following purging solutions with argon or nitrogen for 20–30 min to ensure deoxygenation^11,12,37,44–46^. Diethylenetriamine-pentaacetic-acid (DTPA), a metal chelator (10^–4^ M), prevents auto-oxidation of HS^-^ in aerobic solutions, which in the absence of DTPA is about 50% in about 3 h at 25ºC^37,47^. In addition, solutions should be kept in the dark on ice as 1e^-^ sulfide oxidation is catalyzed by UV light^11^.

**Figure 5:**
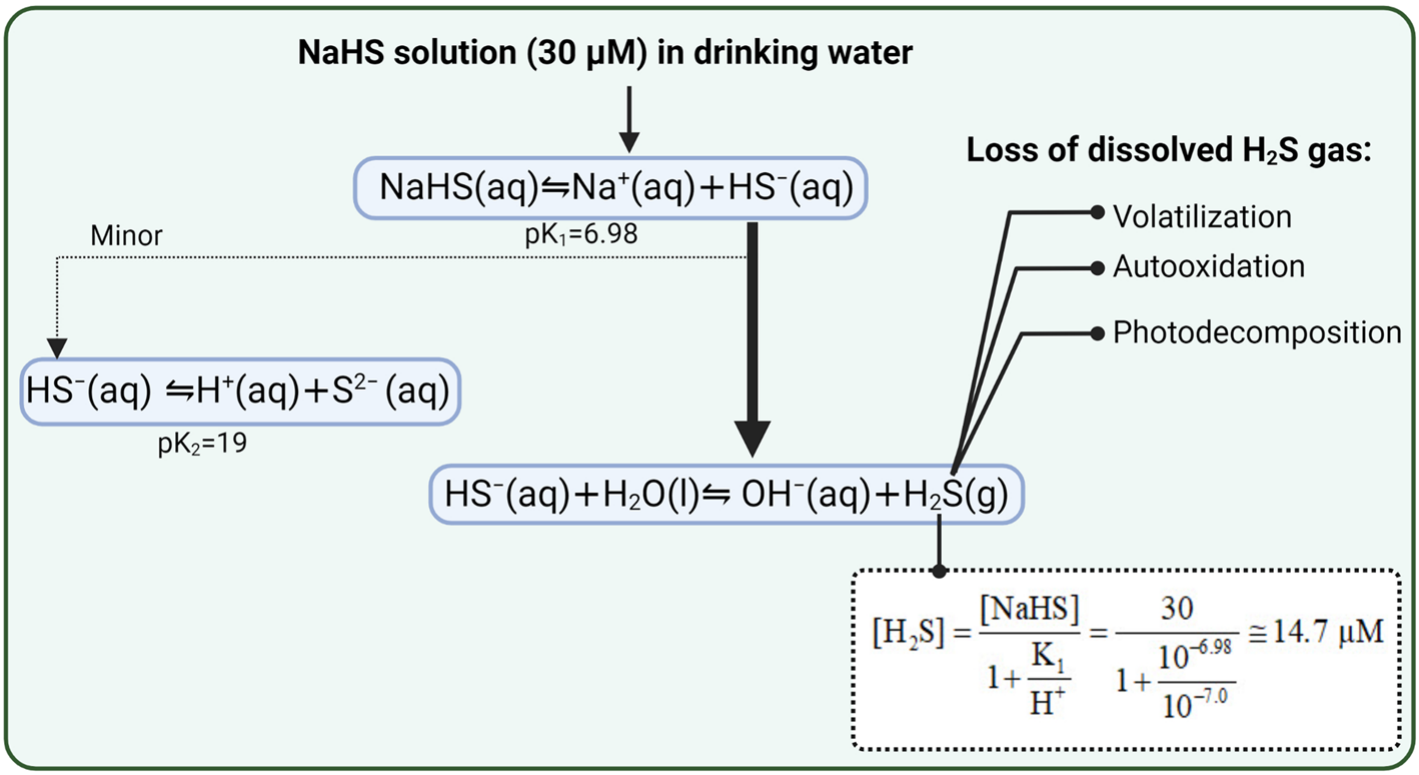
Formation of dissolved H_2_S gas in NaHS solution (30 μM). aq, aqueous; g, gas; l, liquid. All calculations were done assuming water pH = 7.0 and water temperature = 20 °C. Created with BioRender.com.

As shown in Fig. 5, when NaHS is dissolved in water, it ionizes to Na^+^ and HS^-6^; this dissociation depends on pK_1_ of the reaction, which is temperature-dependent: pK_1_ = 3.122 + 1132/T, where T is within 5 to 30º C and used in degrees of Kelvin (K) and K = ºC + 273.15^48^. pK_2_ of HS^-^ is high (pK_2_ = 19), thus, it does not yield S^2-^ or minimal amounts of S^2-^ produced at pH < 9^6,49^. Instead, HS^−^ acts as a base and accepts H^+^ from the H_2_O molecule, which acts as an acid and is converted to H_2_S and OH^−^.

Despite evidence that NaHS solutions are not stable, some animal studies used NaHS solutions in drinking water as an H_2_S-donating compound^15–26^, with the duration of intervention ranging from 1 to 21 weeks (Table 2). These studies refreshed NaHS solutions every 12^15,17,18,24,25^ or 24^19–23^ h. Our results indicate that due to H_2_S loss from the NaHS solution, rats are not exposed to a stable concentration of the drug, and a large fluctuation in the content of NaHS in the drinking water of rats occurs over 12 or 24 h (see Fig. 2). Two of these studies reported that H_2_S content in water was stable for over 24 h^22^ or only 2–3% H_2_S loss was observed over 12 h^15^, but they did not provide supporting data or details of the measurement. Two studies stated that the small aperture of water bottles minimizes H_2_S evaporation^15,19^. However, our results showed that this only retards H_2_S loss from the inside of the water bottle for 2 h, not 12–24 h. In two studies, it has been stated that we presumed that NaHS content in the drinking water was not changed as we did not see changes in water coloring; thus, oxidation of H_2_S due to air is not significant^19,20^. It is a surprise that such a subjective method is used for assessing the stability of NaHS in water instead of measuring its concentration over time.

**Table 2:** Summary of studies that administrated NaHS in drinking water in rodents.

Study	Species	NaHS Concentration (μM)	Refreshment of NaHS solution (hours)	Study duration (weeks)	Temperature (°C)	Reference
Sen et al. 2009	Mouse	30	NR	8	NR	^16^
Sen et al. 2010	Mouse	30	12	8	NR	^15^
Mishra et al. 2010	Mouse	30	24	8–10	22–24	^23^
Givvimani et al. 2011	Mouse	30	24	6	NR	^19^
Qipshidze et al. 2012	Mouse	30	24	4	NR	^20^
Pozsgai et al. 2015	Mouse	~ 400	24	1	24–25	^21^
Askari et al. 2018	Rat	30	12	8	20–22	^17^
Askari et al. 2018	Rat	30	12	8	20–22	^24^
Shirazi et al. 2018	Rat	30	12	8	20–22	^26^
Lee et al. 2018	Mouse	30	24	21	NR	^22^
Lorian et al. 2020	Rat	30	12	8	18–26	^25^
Lorian et al. 2020	Rat	30	12	8	18–26	^18^

*NR* not reported.

H_2_S loss from NaHS solutions is pH- and temperature-dependent. Dissolving NaHS in water produces an alkaline solution^50^, as observed in our study. The production of dissolved H_2_S gas, following the dissolution of NaHS in water, is pH-dependent^6^. The lower the pH solution, the larger the fraction of NaHS in the form of the H2S gas molecules, and the more sulfide is lost from aqueous solutions^11^. None of these studies reported the pH of the drinking water used as the solvent for NaHS. According to WHO guidelines that most countries have adopted, the pH value of drinking water should be in the range of 6.5–8.5^51^. The rate of spontaneous H_2_S oxidation increased by about tenfold over this pH range^13^. Dissolving NaHS in the water with this pH range yields the concentration of dissolved H_2_S gas to be between 1 to 22.5 μM, highlighting the importance of controlling water pH before dissolving the NaHS. In addition, the temperature range reported in the studies mentioned above (18–26 °C) causes about 10% changes in concentration of dissolved H_2_S gas in the solution, as a change in the temperature changes pK_1_ and a slight change in pK_1_ has a substantial effect on the fraction of dissolved H_2_S gas^48^. Adding to this issue is the long duration of some studies (5 months)^22^, during which more variability in temperature is expected to occur.

All studies except one^21^ used 30 μM of NaHS solutions in drinking water. To explain the dose used (i.e., 30 μM), some authors stated that NaHS in the aqueous phase produces exactly equal concentrations of H_2_S gas, and since the physiological range of H_2_S is between 10 and 100 μM, this dose is in the physiological range^15,16^. It has also been explained that 30 μM NaHS keeps plasma H_2_S levels in the physiological range, i.e., 5–300 μM^19,20^. Let us consider a concentration of 30 μM of NaHS in water (pH = 7.0, and T = 20 °C), which had been used in some studies to investigate the effects of H_2_S. We can calculate the concentration of dissolved H_2_S gas to be 14.7 μM, which is about 50% of the initial concentration of NaHS. This value is similar to previous calculations by others under the same conditions^13,48^.

In our study, NaHS administration did not change body weight; this finding is in line with other studies in male mice^22,23^ and male rats^18^; however, two studies reported that NaSH restored reduced body weight in nephrectomized rats^24,26^ and other studies did not report an effect of NaSH administration on body weight^15–17,19–21,25^. In addition, in our study, NaSH administration did not affect serum urea and Cr, a finding that aligns with another report^25^.

This study found that NaHS administration in drinking water for 2 weeks did not affect serum total sulfide concentrations in male and female rats. This finding is in line with results reported by Sen et al.^16^ that 8-week treatment with 30 μM NaHS in drinking water did not affect plasma sulfide levels in control rats; however, they reported that this intervention restored decreased plasma H_2_S levels in nephrectomized mice. Lee et al. also reported that 5 months of treatment with 30 μM NaHS in drinking water increased plasma free sulfide levels by about 26% in aged mice^22^. Other studies have not reported circulating sulfide changes following NaHS intervention in drinking water.

Seven studies have reported that they used NaHS from Sigma^15,16,19–23^ but did not provide more details about water of hydration, and five studies did not mention the source of NaHS used for their preparations^17,18,24–26^. NaHS is a hydrated molecule and may have variable waters of hydration, affecting the amount of NaHS needed for preparing a given molar concentration of the solution. For example, in our study, it was as NaHS•1.3 H_2_O. Therefore, the actual concentrations of NaHS in these studies may be lower than those reported.

“How could such a short-lived compound exert such sustained effects?” this question has been asked by Pozsgai et al.^21^, who assessed the effects of NaHS on colitis in mice. These authors hoped that future studies would answer this question and speculated that the NaHS solution might contain more stable polysulfides besides H_2_S and bisulfides that mediated the NaHS effects^21^. Another possibility is that a very low concentration of NaHS remaining in the solution can provide beneficial effects. Indeed, Olson has provided evidence that the micromolar range of H_2_S in the blood is unphysiological, and it should be in the nanomolar range, or it does not exist in blood at all^13^. There is a possibility that H_2_S acts through protein sulfhydration, which is a reversible post-translational modification and affects the function, stability, and localization of the many proteins^52–54^. Indeed, about 10–25% of many liver proteins are sulfhydrated under physiological conditions^53^. Two studies acknowledged the fast decay of NaHS^19,23,^ but surprisingly, they stated that “we controlled the concentration of NaHS in the drinking water by changing the drinking water every day”^23^. One study surprisingly claims that “NaHS is a standard H_2_S donor which is usually used in clinic instead of H_2_S itself”^18^.

According to the discussion above indicating that NaHS is lost from solution by volatilization, oxidation, and photodecomposition, some suggestions have been provided for minimizing H_2_S loss from solution. First, H_2_S evaporation depends on the air–liquid interface^13^ and pH of the solution^11^; thus, for minimizing loss from volatilization, the size of the aperture of water bottles can be minimized as much as possible, as suggested previously^15,19^ and pH of water can be adjusted at the upper acceptable range (i.e., 6.5–8.5^51^) to minimize loss from volatilization^11^. Second, spontaneous oxidation of H_2_S is due to exposure to oxygen and the presence of transition metal ions in drinking water^13^, therefore deoxygenating drinking water using argon or nitrogen^44,45^ and using a metal chelator^37,47^ can decrease sulfide oxidation. Third, to prevent the photodecomposition of H_2_S, water bottles can be wrapped in aluminum foils; this practice is also applied to light-sensitive materials, such as streptozotocin^55^. Finally, inorganic sulfide salts (NaHS, Na_2_S, and CaS) can be gavaged instead of dissolving in drinking water as has been reported previously^56–58^; it has been shown that following gavage of radioactive sodium sulfide to rats, it is well absorbed and distributed in almost all tissues^59^. Currently, most studies administer inorganic sulfide salts via the intraperitoneal route; however, this route is minimally used in the clinic^60^. On the other hand, the oral route is the most common and preferred route of drug administration in humans^61^. Thus, we suggest oral gavage for assessing the effects of H_2_S donors in rodents.

As a limitation, we measured sulfide levels in aqueous solutions and serum using the MB method. Methods for measurement of sulfide include iodometric, spectrophotometric, electrochemical (potentiometric, galvanic, coulometric, and amperometric), and chromatographic (gas chromatography and HPLC), of which spectrophotometric MB method is the most common approach^62^. A limitation of the MB method for measuring H_2_S levels in biological samples is that it measures all sulfur species rather than free H_2_S^63^ because it is done under acidic conditions, which extracts sulfur from biological sources^64^. However, according to the American Public Health Association, MB is the standard method for measuring aqueous sulfide^65^. Therefore, this limitation does not affect our main result on the instability of the NaHS-containing solution. In addition, our study's sulfide measurement recovery was 91% and 93% for NaHS-containing water and serum samples, respectively. These values are in line with the previously reported range (77–92)^66^ and indicate reasonable accuracy of the assay^42^. As a strength, we used both sexes of rats according to the recommendation of the National Institutes of Health (NIH) to avoid over-reliance on male-only animal research in preclinical studies^67^ and include both sexes when possible^68^. Others have emphasized this issue^69–71^.

In conclusion, the findings of this study indicate that NaHS solution prepared in drinking water cannot be used for H_2_S donation as the solution is not stable. This route of administration exposes animals to variable and lower-than-expected amounts of NaHS; data derived, therefore, may not be translated to humans.

### Supplementary Information

Supplementary Figures.

## Data Availability

The datasets used and/or analyzed during the current study are available from the corresponding author upon reasonable request.

## References

[1] Szabo C (2018). A timeline of hydrogen sulfide (H2S) research: From environmental toxin to biological mediator. Biochem. Pharmacol..

[2] Abe K, Kimura H (1996). The possible role of hydrogen sulfide as an endogenous neuromodulator. J. Neurosci..

[3] Cirino G, Szabo C, Papapetropoulos A (2023). Physiological roles of hydrogen sulfide in mammalian cells, tissues, and organs. Physiol. Rev..

[4] Dillon KM, Carrazzone RJ, Matson JB, Kashfi K (2020). The evolving landscape for cellular nitric oxide and hydrogen sulfide delivery systems: A new era of customized medications. Biochem. Pharmacol..

[5] Sun X (2017). A long-term and slow-releasing hydrogen sulfide donor protects against myocardial ischemia/reperfusion injury. Sci. Rep..

[6] Sitdikova GF, Fuchs R, Kainz V, Weiger TM, Hermann A (2014). Phosphorylation of BK channels modulates the sensitivity to hydrogen sulfide (H2S). Front. Physiol..

[7] Sitdikova GF, Weiger TM, Hermann A (2010). Hydrogen sulfide increases calcium-activated potassium (BK) channel activity of rat pituitary tumor cells. Pflugers Arch..

[8] Jeddi S (2022). Hydrogen sulfide potentiates the protective effects of nitrite against myocardial ischemia-reperfusion injury in type 2 diabetic rats. Nitric Oxide.

[9] Corvino A (2021). Trends in H2S-donors chemistry and their effects in cardiovascular diseases. Antioxidants.

[10] DeLeon ER, Stoy GF, Olson KR (2012). Passive loss of hydrogen sulfide in biological experiments. Anal. Biochem..

[11] Nagy P (2014). Chemical aspects of hydrogen sulfide measurements in physiological samples. Biochim. Biophys. Acta.

[12] Cline JD (1969). Spectrophotometric determination of hydrogen sulfide in natural waters 1. Limnol. Oceanogr..

[13] Olson KR (2012). A practical look at the chemistry and biology of hydrogen sulfide. Antioxid. Redox Signal..

[14] Bamesberger WL, Adams DF (1969). Improvements in the collection of hydrogen sulfide in cadmium hydroxide suspension. Environ. Sci. Technol..

[15] Sen U (2010). Hydrogen sulfide regulates homocysteine-mediated glomerulosclerosis. Am. J. Nephrol..

[16] Sen U (2009). Hydrogen sulfide ameliorates hyperhomocysteinemia-associated chronic renal failure. Am. J. Physiol. Renal Physiol..

[17] Askari H (2018). Ameliorative effects of hydrogen sulfide (NaHS) on chronic kidney disease-induced brain dysfunction in rats: Implication on role of nitric oxide (NO) signaling. Metab. Brain Dis..

[18] Lorian K (2020). Long-term NaHS administration reduces oxidative stress and apoptosis in a rat model of left-side varicocele. Andrologia.

[19] Givvimani S (1985). Hydrogen sulfide mitigates transition from compensatory hypertrophy to heart failure. J. Appl. Physiol..

[20] Qipshidze N, Metreveli N, Mishra PK, Lominadze D, Tyagi SC (2012). Hydrogen sulfide mitigates cardiac remodeling during myocardial infarction via improvement of angiogenesis. Int. J. Biol. Sci..

[21] Pozsgai G, Benkó R, Barthó L, Horváth K, Pintér E (2015). Thermal spring water drinking attenuates dextran-sulfate-sodium-induced colitis in mice. Inflammopharmacology.

[22] Lee HJ (2018). Hydrogen sulfide ameliorates aging-associated changes in the kidney. Geroscience.

[23] Mishra PK, Tyagi N, Sen U, Givvimani S, Tyagi SC (2010). H2S ameliorates oxidative and proteolytic stresses and protects the heart against adverse remodeling in chronic heart failure. Am. J. Physiol. Heart Circ. Physiol..

[24] Askari H (2018). Protective effects of hydrogen sulfide on chronic kidney disease by reducing oxidative stress, inflammation and apoptosis. EXCLI J..

[25] Lorian K, Kadkhodaee M, Kianian F, Abdi A, Seifi B (2020). Administration of sodium hydrosulfide reduces remote organ injury by an anti-oxidant mechanism in a rat model of varicocele. Iran. J. Basic Med. Sci..

[26] Shirazi MK (2019). The role of nitric oxide signaling in renoprotective effects of hydrogen sulfide against chronic kidney disease in rats: Involvement of oxidative stress, autophagy and apoptosis. J. Cell. Physiol..

[27] Reagan-Shaw S, Nihal M, Ahmad N (2008). Dose translation from animal to human studies revisited. Faseb J..

[28] Bahadoran Z, Mirmiran P, Kashfi K, Ghasemi A (2020). Importance of systematic reviews and meta-analyses of animal studies: Challenges for animal-to-human translation. J. Am. Assoc. Lab. Anim. Sci..

[29] Ghasemi A, Jeddi S, Kashfi K (2021). The laboratory rat: Age and body weight matter. Excli J..

[30] Hackam DG, Redelmeier DA (2006). Translation of research evidence from animals to humans. JAMA.

[31] Ahmadi-Noorbakhsh S (2021). Guideline for the care and use of laboratory animals in Iran. Lab. Anim. (NY).

[32] Percie du Sert N, Hurst V, Ahluwalia A (2020). The ARRIVE guidelines 2.0: Updated guidelines for reporting animal research. J. Physiol..

[33] Bettelheim FA, Brown WH, Campbell MK, Farrell SO, Torres O (2020). Introduction to General, Organic and Biochemistry.

[34] Khankari RK, Grant DJ (1995). Pharmaceutical hydrates. Thermochim. Acta.

[35] Festing MF (2018). On determining sample size in experiments involving laboratory animals. Lab. Anim..

[36] Bate ST, Clark RA (2014). The Design and Statistical Analysis of Animal Experiments.

[37] Shen X (2011). Measurement of plasma hydrogen sulfide in vivo and in vitro. Free Radic. Biol. Med..

[38] Cao X (2018). A review of hydrogen sulfide synthesis, metabolism and measurement: Is modulation of hydrogen sulfide a novel therapeutic for cancer?. Antioxid. Redox Signal..

[39] Ubuka T (2002). Assay methods and biological roles of labile sulfur in animal tissues. J. Chromatogr. B Analyt. Technol. Biomed. Life Sci..

[40] Olson KR (2009). Is hydrogen sulfide a circulating "gasotransmitter" in vertebrate blood?. Biochim. Biophys. Acta.

[41] Geng B (2004). Endogenous hydrogen sulfide regulation of myocardial injury induced by isoproterenol. Biochem. Biophys. Res. Commun..

[42] Gonzalez AG, Herrador MA, Asuero AG (1999). Intra-laboratory testing of method accuracy from recovery assays. Talanta.

[43] Moest R (1975). Hydrogen sulfide determination by the methylene blue method. Anal. Chem..

[44] Zhao F (2018). Hydrogen sulfide alleviates placental injury induced by maternal cigarette smoke exposure during pregnancy in rats. Nitric Oxide.

[45] Fogo JK, Popowsky M (1949). Spectrophotometric determination of hydrogen sulfide: Methylene blue method. Anal. Chem..

[46] Stasko A, Brezova V, Zalibera M, Biskupic S, Ondrias K (2009). Electron transfer: a primary step in the reactions of sodium hydrosulphide, an H2S/HS(-) donor. Free Radic. Res..

[47] Hughes MN, Centelles MN, Moore KP (2009). Making and working with hydrogen sulfide: The chemistry and generation of hydrogen sulfide in vitro and its measurement in vivo: A review. Free Radic. Biol. Med..

[48] Broderius SJ, Smith LL (1977). Direct determination and calculation of aqueous hydrogen sulfide. Anal. Chem..

[49] Myers RJ (1986). The new low value for the second dissociation constant for H2S: Its history, its best value, and its impact on the teaching of sulfide equilibria. J. Chem. Educ..

[50] Medvedeva M, Gorelik A (2007). Dangers from improper use of aluminum tank cars. Chem. Petrol. Eng..

[51] Shah A, Arjunan A, Baroutaji A, Zakharova J (2023). A review of physicochemical and biological contaminants in drinking water and their impacts on human health. Water Sci. Eng..

[52] Paul BD, Snyder SH (2012). H_2_S signalling through protein sulfhydration and beyond. Nat. Rev. Mol. Cell Biol..

[53] Mustafa AK (2009). H_2_S signals through protein S-sulfhydration. Sci. Signal..

[54] Sen N (2017). Functional and molecular insights of hydrogen sulfide signaling and protein sulfhydration. J. Mol. Biol..

[55] Ghasemi A, Jeddi S (2023). Streptozotocin as a tool for induction of rat models of diabetes: A practical guide. EXCLI J..

[56] Velázquez-Moyado JA (2015). Gastroprotective effect of diligustilide isolated from roots of Ligusticum porteri coulter & rose (Apiaceae) on ethanol-induced lesions in rats. J. Ethnopharmacol..

[57] Chávez-Piña AE, Tapia-Álvarez GR, Navarrete A (2010). Inhibition of endogenous hydrogen sulfide synthesis by PAG protects against ethanol-induced gastric damage in the rat. Eur. J. Pharmacol..

[58] Medeiros JVR (2009). Hydrogen sulfide prevents ethanol-induced gastric damage in mice: Role of ATP-sensitive potassium channels and capsaicin-sensitive primary afferent neurons. J. Pharmacol. Exp. Ther..

[59] Dziewiatkowski DD (1945). Fate of ingested sulfide sulfur labelled with radioactive sulfur in the rat. J. Biol. Chem..

[60] Al Shoyaib A, Archie SR, Karamyan VT (2020). Intraperitoneal route of drug administration: Should it be used in experimental animal studies?. Pharm. Res..

[61] Alqahtani MS, Kazi M, Alsenaidy MA, Ahmad MZ (2021). Advances in oral drug delivery. Front. Pharmacol..

[62] Lawrence NS, Davis J, Compton RG (2000). Analytical strategies for the detection of sulfide: A review. Talanta.

[63] Jeddi S, Gheibi S, Kashfi K (2020). Dose-dependent effects of long-term administration of hydrogen sulfide on myocardial ischemia-reperfusion injury in male Wistar rats: Modulation of RKIP, NF-κB, and oxidative stress. Int. J. Mol. Sci..

[64] Hartle MD, Pluth MD (2016). A practical guide to working with H 2 S at the interface of chemistry and biology. Chem. Soc. Rev..

[65] Reese BK, Finneran DW, Mills HJ, Zhu M-X, Morse JW (2011). Examination and refinement of the determination of aqueous hydrogen sulfide by the methylene blue method. Aquat. Geochem..

[66] Richardson CJ, Magee EA, Cummings JH (2000). A new method for the determination of sulphide in gastrointestinal contents and whole blood by microdistillation and ion chromatography. Clin. Chim. Acta.

[67] Clayton JA, Collins FS (2014). Policy: NIH to balance sex in cell and animal studies. Nature.

[68] Beery AK, Zucker I (2011). Sex bias in neuroscience and biomedical research. Neurosci. Biobehav. Rev..

[69] McCullough LD (2014). NIH initiative to balance sex of animals in preclinical studies: Generative questions to guide policy, implementation, and metrics. Biol. Sex Differ..

[70] Ritz SA (2014). First steps for integrating sex and gender considerations into basic experimental biomedical research. FASEB J..

[71] Karp NA, Reavey N (2019). Sex bias in preclinical research and an exploration of how to change the status quo. Br. J. Pharmacol..

