# Supplementary material for: Dissolving sodium hydrosulfide in drinking water is not a good source of hydrogen sulfide for animal studies
Source: Sci Rep. 2023 Dec 9;13:21839. doi: 10.1038/s41598-023-49437-y (PMC10710449; doi:10.1038/s41598-023-49437-y)
Supplement: Supplementary file 1 — Supplementary Figures. [file 41598_2023_49437_MOESM1_ESM.docx]

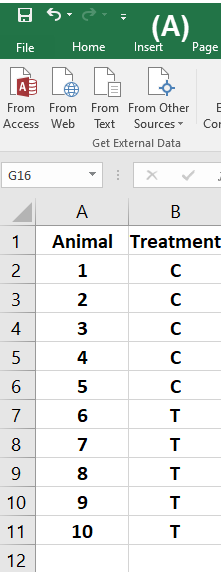

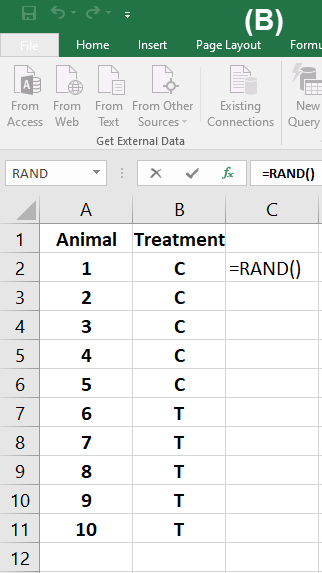

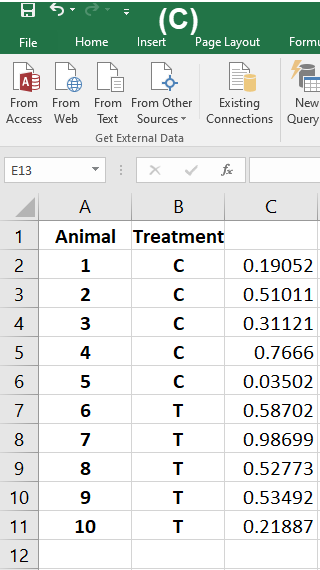

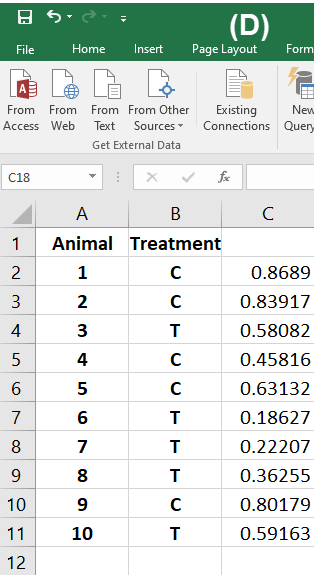


**Supplementary Figure 1.** Randomization of rats using the random function of the Excel software. In this study, 10 rats from each sex were randomized to 2 groups: control (C), and NaSH-treated (NT). **(A)** Write animal ID in column A and study groups in column B in a nonrandom order; **(B)** Use Excel’s random number generator to create a random number in column C2; formula = RAND(); **(C)** Drag the formula in C2 down to fill the remaining cells C3 to C11. This creates a list of 10 random numbers between 0 and 1; **(D)** Select the group column (column B) and the random numbers column (column C) and use Excel's Data → Sort command to sort column B by column C. We have now randomized rats into two groups.

**Supplementary Figure 2.** Standard curve of sulfide measurement using methylene blue method. Data are mean±SEM (n=9).
